# SHH and Notch regulate SOX9+ progenitors to govern arcuate POMC neurogenesis

**DOI:** 10.3389/fnins.2022.855288

**Published:** 2022-08-11

**Authors:** Elsie Place, Elizabeth Manning, Dong Won Kim, Arisa Kinjo, Go Nakamura, Kyoji Ohyama

**Affiliations:** ^1^School of Biosciences, The University of Sheffield, Sheffield, United Kingdom; ^2^Solomon H. Snyder Department of Neuroscience, Johns Hopkins University School of Medicine, Baltimore, MD, United States; ^3^Department of Histology and Neuroanatomy, Tokyo Medical University, Tokyo, Japan

**Keywords:** POMC, hypothalamus, neurogenesis, SOX9, EMT, SHH, Notch

## Abstract

Pro-opiomelanocortin (POMC)-expressing neurons in the hypothalamic arcuate nucleus (ARC) play key roles in feeding and energy homoeostasis, hence their development is of great research interest. As the process of neurogenesis is accompanied by changes in adhesion, polarity, and migration that resemble aspects of epithelial-to-mesenchymal transitions (EMTs), we have characterised the expression and regulation within the prospective ARC of transcription factors with context-dependent abilities to regulate aspects of EMT. Informed by pseudotime meta-analysis of recent scRNA-seq data, we use immunohistochemistry and multiplex *in situ* hybridisation to show that SOX2, SRY-Box transcription factor 9 (SOX9), PROX1, Islet1 (ISL1), and SOX11 are sequentially expressed over the course of POMC neurogenesis in the embryonic chick. Through pharmacological studies *ex vivo*, we demonstrate that while inhibiting either sonic hedgehog (SHH) or Notch signalling reduces the number of SOX9+ neural progenitor cells, these treatments lead, respectively, to lesser and greater numbers of differentiating ISL1+/POMC+ neurons. These results are consistent with a model in which SHH promotes the formation of SOX9+ progenitors, and Notch acts to limit their differentiation. Both pathways are also required to maintain normal levels of proliferation and to suppress apoptosis. Together our findings demonstrate that hypothalamic neurogenesis is accompanied by dynamic expression of transcription factors (TFs) that mediate EMTs, and that SHH and Notch signalling converge to regulate hypothalamic cellular homoeostasis.

## Introduction

Hypothalamic pro-opiomelanocortin (POMC)-expressing neurons in the arcuate nucleus (ARC) play key roles in regulating metabolism and body weight. POMC neurons respond to adipose-derived leptin by signalling to “satiety neurons” expressing the melanocortin receptor, ultimately inhibiting food intake and promoting energy expenditure (Lee and Blackshaw, 2014; Anderson et al., 2016; Quarta et al., 2020). Studies of POMC neurogenesis may therefore lead to the identification of molecular mechanisms that help to prevent metabolic syndrome (Surbhi et al., 2021).

The ARC develops in the anterotuberal region of the hypothalamus and contains a multitude of neuronal subtypes, classically distinguished by their respective complement of neurotransmitters, including POMC (Ohyama et al., 2005; McNay et al., 2006; Saper and Lowell, 2014; Burbridge et al., 2016; Schredelseker and Driever, 2020). Arc neuronal subsets have been described in increasingly finer detail, thanks in part to single-cell RNA-sequencing (scRNA-seq) studies conducted at varying stages of *in utero* and adult development (Campbell et al., 2017; Huisman et al., 2019; Kim et al., 2020; Romanov et al., 2020; Yu et al., 2021). Such work has confirmed some of the key signalling ligands and transcription factors (TFs) underlying the development of ARC POMC neurons, including the TFs ASCL1, NGN3, PRDM12, TBX3, and NK2 Homeobox 1 (NKX2-1) (McNay et al., 2006; Pelling et al., 2011; Bedont et al., 2015). One key TF involved in both the specification of POMC neurons, and continued POMC gene expression, is Islet1 (ISL1) (Lee et al., 2016). POMC neurogenesis continues throughout the lifetime, and ISL1 is also essential for the anorexigenic function of POMC neurons in adult (Nasif et al., 2015), through largely unknown mechanisms (Toda et al., 2017).

The hypothalamus is induced by sonic hedgehog (SHH) (Szabó et al., 2009; Haddad-Tóvolli et al., 2015; Corman et al., 2018) and regionalised by differential WNT/BMP activity, WNT inhibition being necessary for anterior-tuberal hypothalamic fates including POMC neurons (Bedont et al., 2015; Newman et al., 2018). Subsequently, SHH contributes to hypothalamic neurogenesis, as loss of SHH from the hypothalamus in the E10 mouse leads to a loss of *POMC* (Shimogori et al., 2010). This may also hold for the chick, as SHH has been shown to induce ISL1 in the ventral forebrain (Ericson et al., 1995). The Notch signalling pathway also governs POMC neurogenesis: conditional knock-out of the Notch co-factor, Rbpjκ, from the hypothalamus leads to an increase in POMC neurons at the expense of hypothalamic progenitors, while constitutive activation of Notch gives the reverse phenotype (Aujla et al., 2013). Such a pathway may operate also in chick: Notch inhibition causes premature neuronal differentiation in the anterior hypothalamus of both chick and mouse, marked by upregulation of proneural factors including *ASCL1*, *NEUROG1*, and *NHLH1/2* (Ratié et al., 2013; Ware et al., 2016). As yet, however, the point(s) at which SHH and Notch may converge to govern the POMC differentiation programme remains unclear.

The profound transcriptional changes that occur during the developmental trajectory of a neuron are accompanied by correspondingly large phenotypic alterations. Early neural stem and progenitor cells are closely packed within an epithelial monolayer, the neuroepithelium, which gives rise to the apically located neurogenic ventricular zone (VZ) later in development. Neural stem cells (NSCs), in the form of radial glia, reside at the VZ, but change their polarity, adhesive properties and morphology as they delaminate and migrate outward to become intermediate zone (IZ) progenitor cells, and then differentiate into post-mitotic neurons at the outer mantle zone (MZ). This process therefore bears hallmarks of an epithelial-to-mesenchymal transition (EMT) (Singh and Solecki, 2015). Indeed, in processes likened to EMT, Scratch1 and Scratch2, members of the Snail superfamily of core TFs regulating EMT (EMT-TFs), promote delamination and migration of neurons in the mouse neocortex (Itoh et al., 2013); and the core EMT-TF Zeb1 carries related functions in spinal cord astroglia and cerebellar granule neuron progenitors (Ohayon et al., 2016; Singh et al., 2016).

Aside from the small group of core EMT-TFs, many other TFs have been found to have context-dependent abilities to control aspects of EMT (Yang et al., 2020; Debnath et al., 2022). These include several TFs expressed in the developing hypothalamus preceding or during neurogenesis, including ISL1 itself (Brønnum et al., 2013). We therefore set out to characterise the tuberal hypothalamic expression of several TFs linked to EMT among other processes, namely PROX1 (Dadras et al., 2008; Petrova et al., 2008; Elsir et al., 2012; Lu et al., 2012), SRY-Box transcription factor 9 (SOX9) (Akiyama et al., 2004; Cheung et al., 2005; Sakai et al., 2006), SOX11 (Shepherd et al., 2016; Xiao et al., 2020), plus ISL1, and ZEB1. The early neural marker SOX2 was also studied, itself also an EMT regulator (Mandalos et al., 2014; He et al., 2017). The chick embryo was used due to its unparallelled suitability for studying early hypothalamic development (Kim et al., 2022). From our immunostaining results, and backed up by analysis of a recent chicken scRNA-seq dataset (Kim et al., 2022), we infer the sequential expression of SOX2, SOX9, PROX1, ISL1, and SOX11 in the anterior hypothalamus over the course of POMC neurogenesis. By perturbing SHH and Notch signalling in an *ex vivo* assay, we demonstrate that both pathways are required for the maintenance of SOX9+ cycling VZ progenitors upstream of POMC neuronal differentiation. Our findings indicate that SOX9+ hypothalamic VZ progenitors are a key intermediary progenitor cell type, governing cellular homoeostasis, including POMC neurogenesis, in the hypothalamus.

## Materials and methods

### Tissue collection

Fertilised eggs (Henry Stewart & Co., Norfolk, United Kingdom; Poultry 3M, Aichi, Japan) were used according to relevant regulatory standards (The University of Sheffield; Tokyo Medical University). All embryos were staged according to Hamburger and Hamilton (1951).

### Immunolabelling

Chick embryos (*n* = 5–8; each stage) were examined as described previously (Ohyama et al., 2005). Embryos and explants were fixed in 4% Paraformaldehyde for 2 h at 4°C, washed in phosphate buffered saline (PBS), and transferred to 30% sucrose overnight. Samples were mounted in OCT compound and cryosectioned, washed with PBS and primary antibodies were applied overnight at 4°C in blocking buffer (PBS, 1% heat inactivated donkey serum, 0.1% Triton-X-100). Sections were then washed with PBS and incubated in secondary antibody solution (1:1000 in blocking buffer) for 45 min at room temperature. Sections were mounted in Vectashield with DAPI. Antibodies used were: 5E1 anti-SHH mAb (1:50); anti-ISL1 (4D5; 1:50); ISL1 polyclonal antibody (a gift of H. Edlund); Kyo2-60 anti-NKX2-1 rabbit polyclonal antibody (1:1000) (Ohyama et al., 2005); anti-p57 (Sigma-Aldrich, Darmstadt, Germany, P0357, 1:1000); anti-POMC (1:2500, gift from H. Kawano); anti-PROX1 (R&D, AF2727, 1:1000); anti-SOX2 (R&D, AF2018, 1:1000); anti-SOX9 (R&D systems, Minnesota, United States, AF3075, 1:1000); anti-SOX11 (Millipore, Burlington, Massachusetts, United States, ABN105, 1:1000); anti-TUJ1 (Covance, New Jersey, United States, 1:1000); anti-ZEB1 (Sigma, HPA027524, 1:1000); anti-Phospho Histone H3 (Ser10) (Millipore, 06-570, 1:1000); anti-BLBP (Millipore, ABN14, 1:1000). Secondary antibodies were Alexa fluor-488, -555, and -647-conjugated donkey anti-IgG. Z-stacks were taken using a Zeiss LSM700 and maximum intensity projections were processed with Adobe Photoshop.

### Hybridisation chain reaction

Chick embryos (*n* = 4–5) were fixed overnight at 4°C in 4% Paraformaldehyde. Whole mount HCR was performed on isolated heads according to the manufacturer’s instructions for whole mount chicken embryos (Molecular Instruments, Inc., Los Agneles, United States), using buffers purchased from the manufacturer. Custom probe sets for *SHH* (NM_204821.1), *POMC* (XM_015285103.2), *ISL1* (NM_205414.1), *PROX1* (NM_001005616.1), *GLI1*, *PTCH1* (NM_204960.2), *HES5* (NM_001012695.1), *DLL1* (NM_204973.2), and *ELAVL4* (NM_204830.1) were designed by Molecular Instruments, Inc. and used at 1:50. Probes were triple-plexed and detected using paired amplifiers conjugated to Alexa Fluor-488, -546, and -647. Amplifiers were added at 1:50 and left to bind overnight, before washing and equilibrating in sucrose. 15 μm thick cryosections were imaged using a Zeiss Apotome or an Olympus VS200 slide scanner. After imaging, selected HH14, HH18, and HH21 samples (stained for *SHH*, *PTCH1*, *GLI1*) were equilibrated for 5 min in DNaseI buffer, then stripped by digestion in DNaseI (Cat no. 04716728001, Roche, Basel, Switzerland; 1:50 dilution) for 4 h, washed three times in 2× SSC/30% formamide and three times in 2× SSC, (all steps at 37°C). A second round of staining (*HES5*, *DLL1*, and *ELAVL4*) was then performed according to the manufacturer’s instructions for fresh or frozen tissue sections, omitting the sample preparation stage and resuming from the detection stage. Images from different rounds of HCR were manually aligned as TIFs in Adobe Photoshop.

### Colourimetric *in situ* hybridisation

Explants were fixed overnight at 4°C in 4% Paraformaldehyde and were dehydrated in methanol overnight at −20°C, then rehydrated through a methanol/PBS series and incubated at 68°C for 1 h in hybridisation buffer (50% formamide/50% 2× SSC). The buffer was replaced by 1 μg of antisense DIG-labelled mRNA probe in 1 ml of hybridisation buffer, and incubated at 68°C overnight. Explants were then washed and pre-blocked in 10% HINGS in TBST for 90 min, then transferred to blocking buffer containing anti-digoxigenin antibody (1:2000) at 4°C overnight. Signal was developed in NBT/BCIP (Roche) and imaged using a Leica MZ16F microscope.

### Explant culture

Embryos were dissected in cold L15 medium (Gibco-BRL) and treated with Dispase I to facilitate neuroectoderm isolation (Cat No. 4942086001, Roche; 1 mg/ml in L15 medium at room temperature, 5–15 min). Prospective hypothalamus (pHyp) was dissected from HH6 to HH11 embryos, after early hypothalamic specification has been initiated (Kim et al., 2022). Explants were embedded in collagen gels according to published techniques (Ohyama et al., 2005) and cultured for 2 or 4 days in DMEM/F12 with Mito serum (BD, New Jersey, United States) and Glutamax (GIBCO). Cyclopamine, Sigma-Aldrich; Darmstadt, Germany, (C4116) was added to the medium at 480 nM in 0.1% DMSO and N-[N-(3,5-Difluorophenacetyl)-L-alanyl]-S-phenylglycine t-butyl ester (DAPT) (Sigma 565784) at 20 μM in 0.1% DMSO. Area measurements were taken for regions staining positively for the markers studied, using manual thresholding and the Measure function in FIJI. SOX2/SOX9 co-expression was measured using colour thresholding. Briefly, Leica imaging files were opened in FIJI and background staining was eliminated by adjusting the colour balance for each channel individually. Red and green channels were selected in composite mode, and image type was set to RGB. Yellow/orange pixels were selected using the Hue sliders. All markers were expressed as a percentage of total DAPI-positive area. Unpaired *t*-tests were run on GraphPad Prism 9, and *p* < 0.05 was taken as significant. Mean ± SEM are plotted. *n* in figure legends indicates the number of sections analysed per condition, which in all cases come from a minimum of three different explants.

### EdU and death assays

Explants cultured for 2 or 4 days were incubated for 45 min in 20 μM EdU, then immediately fixed and sectioned as per above. 5-ethynyl-2’-deoxyuridine (EdU) staining was performed using the Click-iT Plus EdU imaging kit (Thermo Fisher, Massachusetts, United States), and TUNEL staining using the *in situ* cell death detection kit, TMR red (Roche), according to the manufacturer’s instructions.

### Single-cell RNA-sequencing data analysis

Genes of interest were plotted from a previously published pseudotime analysis of tuberal hypothalamic development (Kim et al., 2022; their Figure 5A, C1→C4→C6 trajectory). The pseudotime was generated from scRNA-seq data of chicken hypothalamus between stages HH8 and HH21. Expression levels of each gene were normalised to their highest and lowest values in the trajectory, i.e., dark blue does not necessarily indicate zero/undetectable expression. The number of *ISL1*-, *PROX1*-, *POMC*-, and *SOX9*-positive cells was extracted from the mature tuberal hypothalamic neurons (C6, Figure 5A in Kim et al., 2022) to draw a Venn diagram. See original publication for details of sample collection and data analysis including pseudotime.

## Results

### NKX2-1+/ISL1+ cells differentiate into POMC neurons in the SHH+ basal plate of chick hypothalamus

**FIGURE 1:**
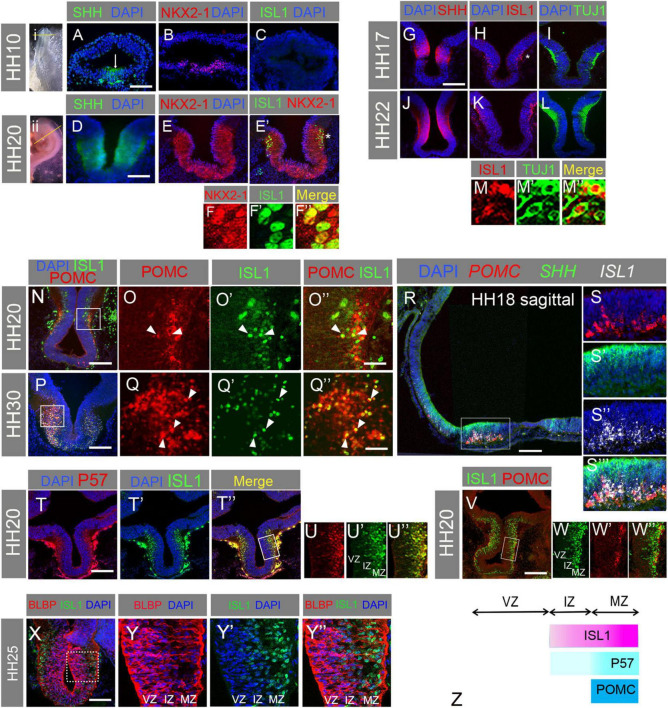
**(A–F”)** Transverse sections of HH10 **(A–C)** and HH20 **(D–E’)** embryos through hypothalamus as indicated in whole mount (i, ii), immunolabelled for SHH, NKX2.1, and ISL1. NKX2-1/ISL1 co-expressing cells can be seen at high magnification in panels **(F–F”)**. Arrow = hypothalamus; * in panel **(E’)** marks the area shown in panel **(F)**. **(G–M”)** Transverse sections of HH17 **(G–I)** and HH20 **(J–L)** embryos through basal hypothalamus, immunolabelled for SHH **(G,J)**, ISL1 **(H,K)**, and TUJ1 **(I,L)**. A high magnification image was taken from an embryo double stained for ISL1 **(M)** and TUJ1 **(M’)** from the region of the hypothalamus indicated by the * in panel **(H)**. Co-expressing cells are observed in panel **(M”)**. **(N–Q”)** Transverse sections of HH20 **(N–O”)** and HH30 **(P–Q”)** embryos through basal hypothalamus, immunolabelled for POMC and ISL1 **(N,P)**. Box in panel **(N)** indicates region shown in panels **(O–O”)**. Box in panel **(P)** indicates region shown in panels **(Q–Q”)**. Arrowheads indicate co-expressing cells. **(R–S”’)** Sagittal section through HH18 hypothalamus processed by HCR to detect mRNA for *POMC*, *SHH*, and *ISL1*. Box in panel **(R)** indicates region shown in panels **(S–S”’)**. **(T–U”)** Transverse sections through HH20 hypothalamus immunolabelled for P57 and ISL1. Box in panel **(T”)** indicates region shown in panels **(U–U”)**. **(V–W”)** Transverse sections through HH20 hypothalamus immunolabelled for ISL1 and POMC. Box in panel **(V)** indicates region shown in panels **(W–W”)**. **(X–Y”)** Transverse sections of HH25 hypothalamus immunolabelled for BLBP and ISL1. Box in panel **(X)** indicates region shown in panels **(Y–Y”)**. **(Z)** Schematic summarising the zonal locations of ISL1, P57, and POMC. VZ, ventricular zone; IZ, intermediate zone; MZ, mantle zone. Scale bars: **(A)** 160 μm; **(D)** 80 μm; **(G)** 160 μm; **(N,P)** 160 μm; **(O,Q)** 40 μm; **(R)** 100 μm; **(T,V,X)** 160 μm.

We first assessed the initial appearance of POMC neurons, relative to differentiating hypothalamic ISL1+ cells. ISL1 protein was not present in the pre-neurogenic SHH+/NKX2-1+ hypothalamus at HH10 (Figures 1A–C), but was readily detected in the SHH+/NKX2-1+ basal hypothalamus by HH20 (Figures 1D–F”). As early as HH17 ISL1+ cells co-expressed TUJ1, a beta-tubulin that marks neurons (Figures 1G–M”). Consistent with previous studies in mice and zebrafish (Nasif et al., 2015; Lee et al., 2016), POMC was present in ISL1+ neurons over HH20-HH30 (Figures 1N–Q”), and indeed we detected *ISL1* and *POMC* mRNA in the *SHH*-positive anterotuberal hypothalamus as early as HH18 (Figures 1R–S”’). Together, this confirms that NKX2-1+/ISL1+ cells differentiate into POMC neurons in the SHH+ basal plate of the chick hypothalamus.

We also monitored protein expression of P57, a cyclin-dependent kinase inhibitor and marker of cell cycle exit and early differentiation. P57 was observed primarily at the MZ, while ISL1 was detected in both the IZ and MZ (Figures 1T–U”). This indicates that ISL1 labels late progenitors and post-mitotic neurons, and that ISL+ cells at the MZ are post-mitotic neurons. Close examination of double labelled HH20 sections furthermore showed that POMC+/ISL1+ cells were located exclusively at the MZ and not the IZ (Figures 1V–W”). Finally we examined expression of ISL1 relative to the radial glial marker BLBP (also known as FABP7). BLBP was detected at high levels in radial glial cell bodies at the VZ, and in radial glial processes that extended to the MZ (Figure 1X). ISL1 was not detected in the BLBP-expressing radial glial cell bodies, and there was only partial overlap in ISL and BLBP expression in the IZ or MZ (Figures 1Y–Y”). This suggests that cells downregulate neuroepithelial/radial glial characteristics as they detach from the apical surface and initiate neurogenesis. These findings are summarised in Figure 1Z.

### Transcription factors expressed early in tuberal neurogenesis are detected in the ventricular zone

**FIGURE 2:**
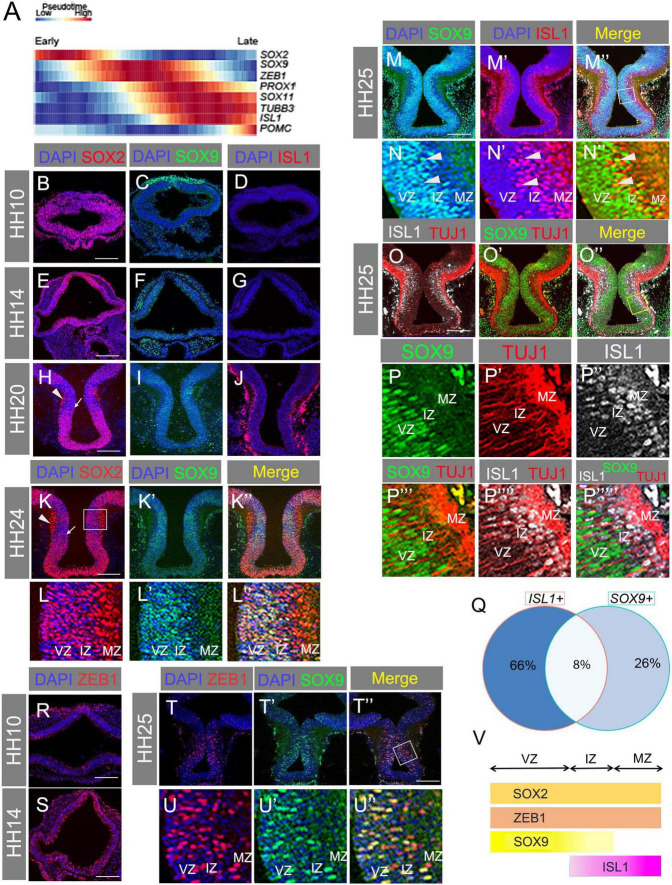
**(A)** Pseudotime trajectory of maturing chicken tuberal hypothalamic cells from HH8 to HH21, showing relative expression levels of the factors indicated. **(B–J)** Transverse sections through basal hypothalamus at HH10, 14, and 20 with antibody labelling for SOX2 **(B,E,H)**, SOX9 **(C,F,I)**, and ISL1 **(D,G,J)**. **(K–L”)** Transverse section through HH24 hypothalamus co-labelled with SOX2 and SOX9 antibodies. Box in panel **(K)** indicates region shown in panels **(L–L”)**. Note that SOX2 expression is reduced in the VZ/IZ, and highest in the MZ in the boxed area in panel **(K)**, which is shown in panels **(L–L”)**. Arrowhead = high MZ expression of SOX2; Arrow = lower VZ/IZ expression of SOX2. **(M–N”)** Transverse section through HH25 hypothalamus co-labelled with antibodies for SOX9 and ISL1. **(M”)** Merged image with box indicating area expanded in panels **(N–N”)**. Arrowheads indicate co-expressing cells. **(O–P””’)** Transverse section through HH25 hypothalamus co-labelled with antibodies for TUJ1, SOX9, and ISL1. **(O”)** Merged image with box indicating area expanded in panels **(P–P””’)**. **(Q)** Venn diagram showing percentage of *ISL1*- and *SOX9*-expressing cells that are single and double positive for these markers. **(R,S)** Transverse section through hypothalamus at HH10 **(R)** and HH14 **(S)** with antibody labelling of ZEB1. **(T–U”)** Transverse section through HH25 hypothalamus co-labelled with antibodies for ZEB1 and SOX9. **(T”)** Merged image with box indicating area expanded in panels **(U–U”)**. **(V)** Schematic summarising the zonal locations of SOX2, ZEB1, SOX9, and ISL1. VZ, ventricular zone; IZ, intermediate zone; MZ, mantle zone. Scale bars: all 160 μm.

Our results suggest that ISL1 is expressed within IZ and MZ cells that have delaminated from the VZ. Of possible relevance, ISL1 has the context-dependent ability to promote EMT and mesenchymal characteristics in epicardial cells (Brønnum et al., 2013). We therefore analysed scRNA-seq data from our recent study (Kim et al., 2022), utilising a pseudotime trajectory of chick tuberal hypothalamic cells (including ARC *POMC*+ neurons) that was generated from a combined dataset of six developmental stages from HH8 to HH20–21. We searched for a selection of TFs previously connected to EMT (see below), in some cases also with known involvement in POMC neurogenesis. Pseudotime analysis suggested that *SOX2* expression peaks early, corresponding to early hypothalamic neuroepithelial stages then subsides as *SOX9* and *ZEB1* upregulate, then increases again toward the end of the trajectory (pale blue) (Figure 2A). Following *SOX9/ZEB1* upregulation, *PROX1*, *SOX11*, and *ISL1* initiate, roughly coincident with *TUBB3* (the gene encoding TUJ1 protein) (Figure 2A). Of the factors analysed, *POMC* is the last to be expressed, in keeping with the observation *in vivo* that POMC is restricted to ISL1+ MZ neurons (Figures 1N–S”’,V–W”).

We sought to validate these results *in vivo* by analysing protein localisation (VZ/IZ/MZ) and co-expression of each marker. SOX2 was found widely in the hypothalamus at all stages examined (HH10–HH24) (Figures 2B,E,H,K,L), although at HH20 and HH24, VZ/IZ expression was weak, relative to MZ expression, in regions of the basal hypothalamus characterised by a well-developed, ISL1+ MZ (see below) (Figures 2L–L”,V). SOX9 was present at the neural tube dorsal midline at HH10, but was not detected in the hypothalamus until HH14 (Figures 2C,F). By HH20–HH25, SOX9 was co-expressed with reduced levels of SOX2 at the VZ/IZ but was not present at the ISL1+, TUJ1+ MZ (Figures 2I,K’–L”). A subset of SOX9+ cells stained positive for ISL1 in the IZ, but not in the VZ or the TUJ1+ MZ (Figures 2M–P””’). Corroborating this, bioinformatics analysis of developing tuberal neurons detected substantial numbers of cells co-expressing *SOX9* and *ISL1* mRNA, with ∼24% of *SOX9*+ cells also containing *ISL1* mRNA and ∼11% of *ISL1*+ cells positive for *SOX9* mRNA (Figure 2Q). This suggests that at least a proportion of *ISL1*+ cells are descended from *SOX9*+ progenitors.

Pseudotime analysis suggested that *ZEB1* expression increased at a similar time to *SOX9* (Figure 2A). This was validated by immunostaining showing little-to-no expression in the HH10 hypothalamus but widespread expression from HH14 (Figures 2R,S). At HH25 ZEB1 was peppered throughout the hypothalamic VZ, IZ, and MZ, co-localising with SOX9 (Figures 2T–U”). In summary, transcription factors expressed early in the pseudotime trajectory of tuberal neurogenesis are detected at early stages of development (HH10, HH14) and/or in the VZ at later stages (Figure 2V).

### Transcription factors expressed later in POMC-expressing neurogenesis are detected in the intermediate zone and mantle zone

**FIGURE 3:**
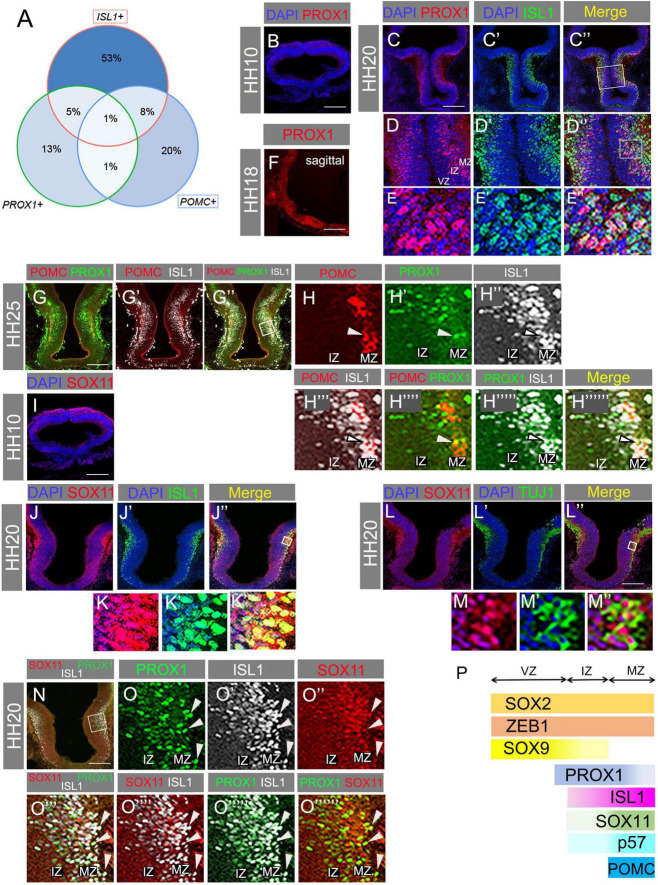
**(A)** Venn diagram showing percentage of ISL1-, PROX1-, or POMC-expressing cells that are single, double, or triple positive for these markers. **(B)** Transverse section through HH10 hypothalamus with antibody labelling for PROX1. **(C–E”)** Transverse section through hypothalamus at HH20 immunolabelled for PROX1 and ISL1. Box in panel **(C”)** indicates area expanded in panels **(D–D”)**. Box in panel **(D”)** indicates area expanded in panels **(E–E”)**. **(F)** Sagittal section showing *PROX1* mRNA detected by HCR at HH18. **(G–H”””)** Transverse section through HH25 hypothalamus with antibody co-labelling for POMC, PROX1, and ISL1. Box in panel **(G”)** indicates region expanded in panels **(H–H”””)**. Arrowheads indicate co-expressing cells. **(I–J”)** Transverse section through HH20 hypothalamus with antibody co-labelling for SOX11 and ISL1. Box in panel **(J”)** indicates region expanded in panels **(K–K”)**. **(K–L”)** Transverse section through HH20 hypothalamus with antibody co-labelling for SOX11 and TUJ1, and merged. Box in panel **(K”)** indicates region expanded in panels **(L–L”)**. **(M)** Transverse sections through HH10 hypothalamus immunolabelled for SOX11. **(N–O”””)** Transverse section through HH20 hypothalamus with antibody co-labelling for SOX11, PROX1, and ISL1. Box in panel **(N)** indicates area expanded in panels **(O–O”””)**. Arrowheads indicate co-expressing cells. **(P)** Schematic summarising the zonal locations of SOX2, ZEB1, SOX9, PROX1, ISL1, SOX11, P57, and POMC. VZ, ventricular zone; IZ, intermediate zone; MZ, mantle zone. Scale bars: all 160 μm.

*PROX1* has previously been identified as a marker of the hypothalamic premammillary nucleus (Kim et al., 2020). However, analysis of developing tuberal neurons from our scRNA-seq dataset reveals that a small number of cells co-express *PROX1*, *ISL1*, and *POMC*, with many more cells double positive for two of the three markers (Figure 3A). As the data only reflects partial coverage of each cell’s transcriptome, the true proportion of co-expressing cells is certainly higher. Our pseudotime analysis also suggests that *PROX1* expression precedes that of *ISL1* during the course of tuberal hypothalamic development (Figure 2A). We sought to validate these results *in vivo* by analysing protein localisation (VZ/IZ/MZ) and co-expression.

Similar to ISL1, PROX1 protein was absent at HH10 (Figure 3B), but present in the basal plate of the hypothalamus at HH20 (Figures 3C–E”). *PROX1* mRNA was also detected in *POMC*+ regions of the anterotuberal hypothalamus (Figure 3F, compare Figure 1R). PROX1+ cells were mostly found at the IZ, but a few scattered cells were also located in the MZ (Figure 3D). Importantly, in the IZ, some PROX1+/ISL1+ double positive cells were observed (Figures 3E–E”), and at HH25, a small number of cells triple positive for PROX1, ISL1, and POMC were found at the IZ-MZ border (Figures 3G–H”””). This validates the pseudotime *PROX1/ISL1/POMC* trajectory (Figure 2A) and *in silico* co-expression analysis (Figure 3A), and demonstrates that these TFs operate in at least one common pathway. Immunolabelling also shows that ISL1 is detected more weakly in PROX1+ IZ cells, and more strongly in MZ cells (Figures 3C–E”).

Our data indicate that PROX1 expression precedes ISL1 expression in at least a subset of cells, and that PROX1+/ISL1+ cells express P57 (Figures 1T–U”), exit cell cycle and differentiate into post-mitotic cells including POMC neurons at the MZ.

As a final TF of interest, we examined SOX11 protein localisation. At HH10, SOX11 was found in the dorsal ectoderm but not in the basal hypothalamus (Figure 3I). At HH20 SOX11+ cells were absent from the IZ but abundant in the MZ, where most co-expressed ISL1 (Figures 3J–K”) and co-localised with TUJ1 (Figures 3L–M”). Hence, SOX11 is expressed in post-mitotic neurons at the MZ. Considering that *SOX11* mRNA levels began to increase at a similar time to *PROX1* (Figure 2A), it is possible that SOX11 protein translation is delayed. Finally, triple labelling of ISL1, PROX1, and SOX11 demonstrated co-expression of all three factors in a subset of cells in the MZ (arrowheads, Figures 3N–O”””). PROX1+ cells in the VZ/IZ were less likely to express SOX11: this likely reflects the earlier expression of PROX1, and possibly also cell type heterogeneity, as some tuberal progenitors are destined to give rise to alternative neuronal subtypes. Overall, the data presented in Figures 2–3 support the sequential expression of the analysed TFs during the course of tuberal hypothalamic (including POMC) neurogenesis: SOX9 at the neurogenic VZ, PROX1 at the VZ/IZ, ISL1 at the IZ/MZ, and SOX11 at the MZ of the SHH+ basal hypothalamus (summarised in Figure 3P).

### SHH and Notch pathways are active in developing basal hypothalamus

We next considered which pathways might regulate the emergence of SOX9-positive progenitors, and their subsequent differentiation. The critical importance of SHH signalling for the development of the basal hypothalamus has been documented in numerous studies (Szabó et al., 2009; Haddad-Tóvolli et al., 2015; Corman et al., 2018), but its effect on SOX9 in this region has not been investigated. It is also long established that Notch signalling is a key regulator of neurogenesis, yet understanding of Notch signalling in the hypothalamus is in its infancy compared to other parts of the central nervous system. We therefore sought to explore the involvement of these pathways on SOX9 progenitors and POMC neurogenesis.

**FIGURE 4:**
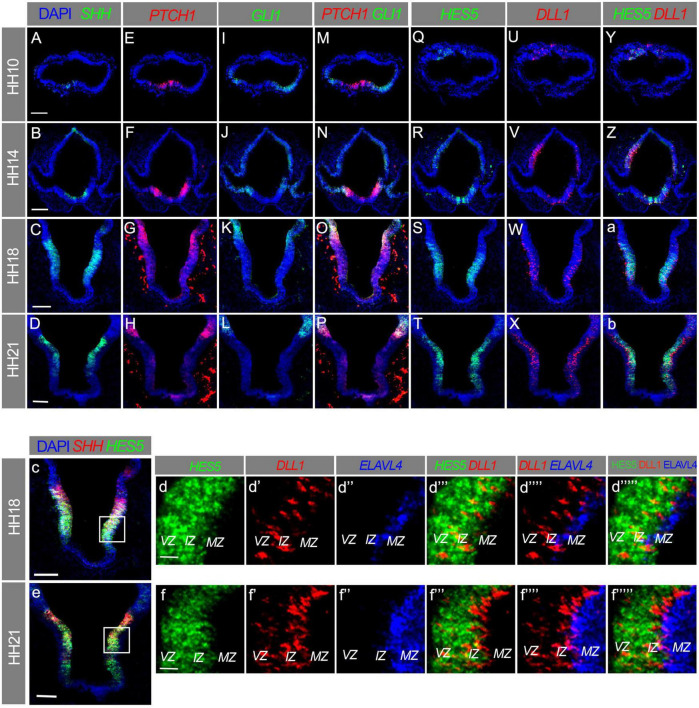
**(A–f””’)** Transverse sections through hypothalamus of HH10 **(A,E,I,M,Q,U,Y)**, HH14 **(B,F,J,N,R,V,Z)**, HH18 **(C,G,K,O,S,W,a,c,d–d””’)**, and HH21 **(D,H,L,P,T,X,b,e,f–f””’)** embryos, *in situ* HCR labelled for *SHH*, *PTCH1*, *GLI1*, *HES5*, *DLL1*, and *ELAVL4*. Boxes in panels **(c,e)** indicate area expanded in panels **(d–d””’,f–f””’)**. VZ, ventricular zone; IZ, intermediate zone; MZ, mantle zone. Scale bars = 100 μm **(A–D,c,e)**, 25 μm **(d,f)**.

*SHH* and its receptor *Patched* (*PTCH1*) are both expressed in the chick basal hypothalamus (Manning et al., 2006). *PTCH1*, and the SHH effector *GLI1* are both induced by SHH and hence provide a readout of SHH activity (Goodrich et al., 1997). We confirmed their (co)expression by multiplex HCR *in situ* hybridisation, and indeed we detected all three in the developing hypothalamus at all stages analysed, from HH10 to HH21 (Figures 4A–P). *SHH* itself was expressed throughout the basal hypothalamus, aside from its ventralmost domain, which becomes SHH-negative between HH14 and HH18 (Figures 4A–D; Manning et al., 2006). *PTCH1* expression was highest in a region immediately dorsal to the *SHH*-expressing domain, which also expresses *GLI1*, but low levels of *PTCH1* were also co-expressed with *SHH* in the basal hypothalamus at HH18 (Figures 4E–P). *PTCH1* was not readily detectable in the basal hypothalamus at HH21, nor was *GLI1* at any stage examined, as observed by others (Figures 4H,L,P; Aglyamova and Agarwala, 2007).

The Notch ligand *DLL1* and Notch target *HES5* were detected in the dorsal anterior neural tube at HH10, whereas the hypothalamus was negative for both at this stage (Figures 4Q,U,Y; Ratié et al., 2013). At HH14, HH18, and HH21, *HES5* was expressed in the VZ/IZ across the majority of the basal plate, encompassing the entire *SHH/PTCH1*-expressing region, while also being absent from the ventralmost hypothalamus (Figures 4Q–T,Y-b). The co-expression of *PTCH1* and *HES5* within the *SHH*-positive basal hypothalamus at HH14 and HH18 indicates that both the SHH and Notch signalling pathways are active in this region (Figures 4F,G,R,S).

*DLL1* was expressed mainly in the IZ with a salt-and-pepper pattern characteristic of this ligand and its receptors, but some *DLL1*+ cells were also present in the VZ (Figures 4V–X,d’,f’). *DLL1* was virtually absent from the MZ, as assessed through co-analysis of *DLL1* and *ELAVL4*, a marker of early differentiating neurons (Figures 4d”’,f”’). As cells expressing the DLL1 ligand send, but do not receive signal (via NOTCH receptors), this data suggests that Notch is active in the VZ and IZ (Figures 4d,f), but is switched off in a subset of IZ cells, and remains silent in more differentiated MZ cells. In sum, this confirms that SHH and Notch pathways are active in the developing hypothalamus. SHH activity precedes that of Notch, which appears by HH14–at which point SOX9-expressing progenitors are also detectable in this tissue (Figure 2F).

### SOX9+ hypothalamic progenitors require SHH

**FIGURE 5:**
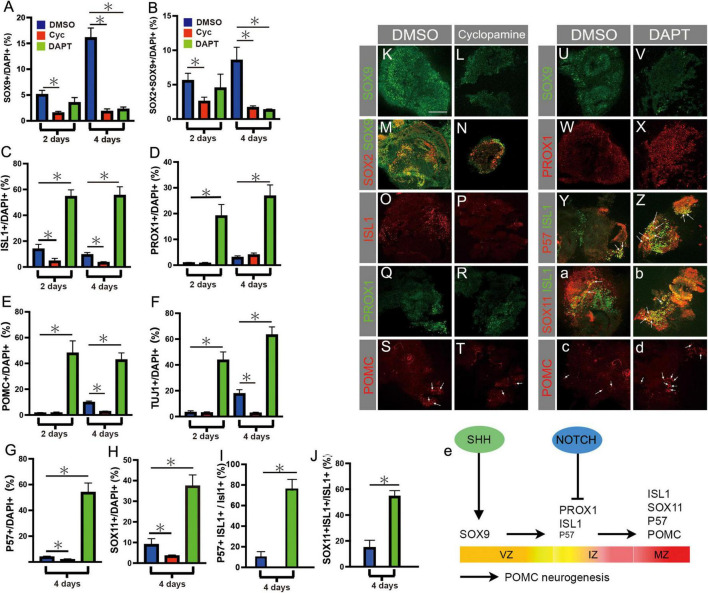
**(A–J)** Quantification of results of *ex vivo* culture experiments conducted on pHyp explants cultured for 2 or 4 days in the presence of DMSO, the SHH inhibitor cyclopamine, or the Notch inhibitor DAPT. Area immunolabelled for SOX9 **(A)**, SOX2 and SOX9 **(B)**, ISL1 **(C)**, PROX1 **(D)**, POMC **(E)**, TUJ1 **(F)**, P57 **(G)**, SOX11 **(H)**, normalised to total DAPI+ area. Area immunolabelled for P57 and ISL1, normalised to total ISL1+ area **(I)**; area immunolabelled for SOX11 and ISL1, normalised to total ISL1+ area **(J)**. Mean ± SEM, *n* = 9–14 (2 days), *n* = 4–13 (4 days). Asterisks indicate statistical significance at *p* < 0.05. **(K–T)** pHyp explants cultured for 2 or 4 days in the presence of DMSO **(K,M,O,Q,S)** or cyclopamine **(L,N,P,R,T)** and immunolabelled for SOX9 **(K,L)**, SOX2 and SOX9 **(M,N)**, ISL1 **(O,P)**, PROX1 **(Q,R)**, and POMC **(S,T)**. Arrows in **(S,T)** indicate POMC+ cells. **(U–d)** pHyp explants cultured for 2 or 4 days in the presence of DMSO **(U,W,Y,a,c)** or the Notch pathway inhibitor DAPT **(V,X,Z,b,d)** and immunolabelled for SOX9 **(U,V)**, PROX1 **(W,X)**, P57 and ISL1 **(Y,Z)**, SOX11 and ISL1 **(a,b)**, and POMC **(S,T)**. Arrows in **(c,d)** indicate POMC+ cells. **(e)** Schematic summarising transcription factor expression over the course of POMC neurogenesis. Proposed roles of SHH signalling in promoting SOX2+/SOX9+ progenitor cells, and of Notch signalling in inhibiting differentiation of IZ PROX1+ISL1+P57+ cells, are indicated. Small letters indicate low abundance of P57 in IZ. Horizontal arrows indicate temporal progression of POMC neurogenesis. VZ, ventricular zone; IZ, intermediate zone; MZ, mantle zone. Scale bars: all 160 μm.

In order to investigate SHH requirements on POMC neurogenesis, we used a previously described *ex vivo* culture assay (Ohyama et al., 2005). Prospective hypothalamic explants were isolated at HH9, and were cultured in the presence of either DMSO (control), or the SHH inhibitor cyclopamine, for 2 or 4 days. Control explants expressed SOX9 and SOX2 at both timepoints, albeit at higher levels at 4 days (Figures 5A,B). This suggests that by 2 days the cultures had reached the equivalent of at least HH14, when SOX9 is detected (Figure 2F) and Notch is active in the basal hypothalamus (Figure 4R). ISL1 levels remained steady between 2 and 4 days, while PROX1, POMC and TUJ1 all increased over this period (Figures 5C–F). This confirms that neuronal differentiation is proceeding in the cultured explants, leading to an increasingly mature cellular composition over time. We next established the efficacy of our experimental conditions, and looked for evidence of SHH pathway inhibition by cyclopamine. *PTCH1* mRNA staining was virtually eliminated by cyclopamine treatment at both 2 and 4 days of culture, indicating successful inhibition (Supplementary Figures 1A–D).

Previous studies in mouse and chick have shown that SHH directs anterotuberal neurogenesis, and we wished to determine whether this occurs through the regulation of SOX9+ VZ cells. Cyclopamine treatment decreased the SOX9+ area by 70 and 89% at 2 and 4 days, respectively, compared to control explants at the same timepoints (Figures 5A,K,L). Similarly, the SOX2+/SOX9+ double positive area was 56% (day 2) and 80% (day 4) less than control (Figures 5B,M,N). Therefore, SHH is required for the development of SOX2+SOX9+ progenitors in cultured hypothalamic explants (Figure 5e).

ISL1+ area was reduced by 67% (day 2) and 64% (day 4) after cyclopamine, relative to controls, but PROX1 levels remained steady (Figures 5C,D,O–R). POMC and TUJ1 areas were low and unaltered in cyclopamine-treated explants at 2 days, but significantly reduced after 4 days, as were both P57 and SOX11 (Figures 5E–H,S,T). Thus, fewer POMC+ neurons were detected in cyclopamine-treated explants, likely a downstream consequence of the shortage of SOX9+ progenitors.

### Notch suppresses PMOC-expressing neuronal differentiation

Previous studies have shown that Notch signalling negatively regulates ASCL1 expression in the hypothalamus, and that ASCL1 is necessary for POMC neuronal development (McNay et al., 2006; Aujla et al., 2013; Ratié et al., 2013; Ware et al., 2016). To further explore the role of Notch signalling in chicken POMC neurogenesis, HH9 pHyp explants were exposed to the Notch inhibitor DAPT and analysed after 2 and 4 days. Efficacy of treatment was confirmed by *in situ* hybridisation for *HES5*, which was detectable in control but not treated explants at both 2 and 4 days (Supplementary Figures 1E–H). No significant change in SOX9+ or SOX2+SOX9+ area was detected at 2 days, but after 4 days, treatment with DAPT led to an 86% decrease in SOX9+ area, and an 89% reduction in SOX2+/SOX9+ area, similar to the effects of cyclopamine (Figures 5A,B,U,V). Therefore, Notch is required for SOX9 expression.

In contrast to the results obtained with cyclopamine, ISL1+ area increased by almost 3-fold (2 days) and 5-fold (4 days) over control levels, while PROX1+ area increased >20-fold and >7.5-fold, respectively (Figures 5C,D,W,X). The late markers P57 and SOX11 were also greatly increased after 4 days of culture (Figures 5G,H). A much higher percentage of the ISL1+ area co-expressed P57 and SOX11, demonstrating that those ISL1+ cells present tended to be more mature as well as greater in number, corresponding to post-mitotic MZ neurons (Figures 5I,J,Y,Z,a,b,1T–U”, 3I–O). The number of POMC neurons was likewise strongly increased at both timepoints (Figures 5E,c,d). Therefore, neurogenic differentiation was elevated and occurred earlier in DAPT-treated explants, indicating that Notch inhibits neurogenic differentiation. This is consistent with earlier reports (Ratié et al., 2013); with the expression of *HES5* throughout the VZ; and with the appearance of *DLL1* in the IZ (Figures 4c–f””’), where markers of early differentiation such as ISL1 and PROX1 are also found (Figures 3H–H””’). These results suggest that Notch helps to maintain SOX9 levels (Figures 5A,B) at least in part by inhibiting the differentiation of SOX9+ neurogenic VZ cells (Figure 5e). In the absence of Notch, precocious differentiation leads to depletion of SOX9+ progenitors, increased generation of PROX1+ VZ/IZ progenitor cells, early cell cycle exit, and more abundant SOX11+/P57+ and POMC+ post-mitotic neurons.

### SHH and Notch are required for cell survival

The above results demonstrate that both SHH and Notch are required for normal SOX9 levels (Figure 5e). Considering that SHH is required to induce SOX2+/SOX9+ neurogenic VZ cells from SOX2+ neuroepithelium in dorsal telencephalon (Scott et al., 2010), SHH could play a similar role in the basal hypothalamus. Is it also possible that SHH regulates proliferation/self-renewal of progenitors, and/or that it acts as a survival factor. Meanwhile, the action of Notch in suppressing neuronal differentiation (Figure 5e) raises the possibility that SOX9+ progenitors become depleted in Notch-inhibited explants via a different mechanism, namely premature differentiation. We therefore analysed markers of proliferation and apoptosis in pHyp explants to shed further light on the effects of pathway manipulation.

**FIGURE 6:**
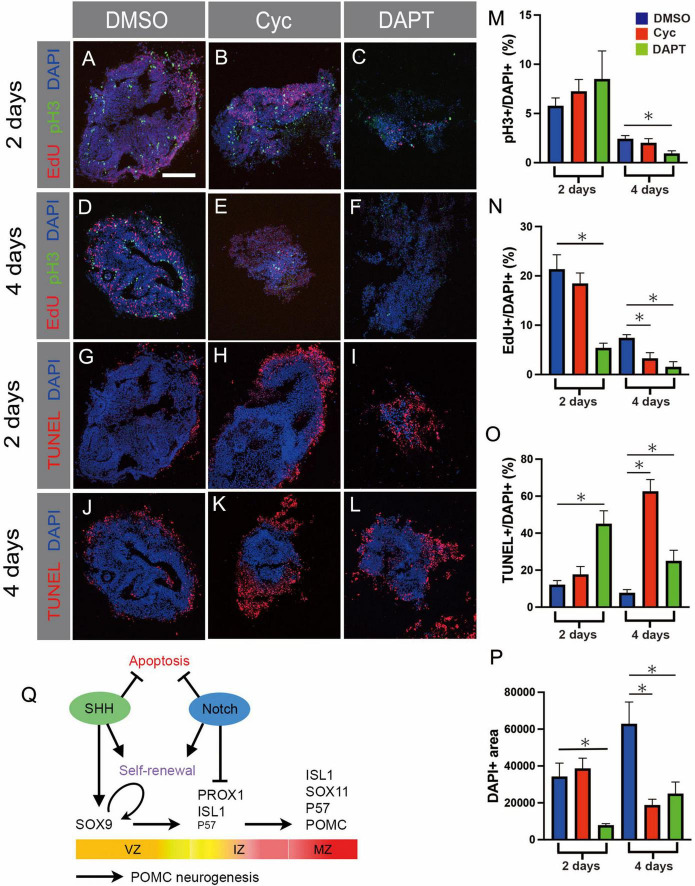
**(A–L)** Prospective hypothalamic (pHyp) explants cultured in the presence of DMSO **(A,D,G,J)**, the SHH inhibitor cyclopamine **(B,E,H,K)**, or the Notch inhibitor DAPT **(C,F,I,L)** for 2 **(A–C,G–I)** or 4 **(D–F,J–L)** days. pH3 immunolabelling **(M)**, EdU detection **(N)**, and the TUNEL assay **(L)** were performed. **(M–P)** Quantification of results of *ex vivo* culture experiments on pHyp explants cultured for 2 or 4 days in the presence of DMSO or cyclopamine or DAPT. Image area staining positively for pH3 **(M)**, EdU **(N)**, or TUNEL signal **(L)**, normalised to total DAPI+ area. Relative DAPI+ area **(P)**. Mean ± SEM, *n* = 12–23 (2 days), *n* = 9–13 (4 days). Asterisks indicate statistical significance at *p* < 0.05. Scale bar: 160 μm **(A)**. **(Q)** Schematic summarising transcription factor expression over the course of POMC neurogenesis. Curved arrow indicates self-renewal of SOX2+/SOX9+ VZ progenitor cells. Proposed roles of SHH signalling in inducing SOX9+ expression; of Notch signalling in inhibiting differentiation of IZ PROX1+ cells; and of both pathways in promoting self-renewal and inhibiting apoptosis, are indicated. Horizontal arrows indicate temporal progression of POMC neurogenesis. VZ, ventricular zone; IZ, intermediate zone; MZ, mantle zone.

Control explants cultured in the presence of DMSO contained proliferating cells, as indicated by both EdU incorporation and phosphorylated Histone 3 (pH3) staining (Figures 6A,D,M,N). Fewer cycling cells were detected after 4 days of culture than 2 days (Figures 6M,N), possibly owing to the higher proportion of post-mitotic neurons in the more mature explants (Figures 5E,F).

No significant changes were detected in proliferative markers or apoptosis after 2 days of SHH inhibition, and total explant area was no different to control (Figures 6B,H,M–P). However, by 4 days EdU incorporation was significantly reduced, and an >8-fold increase in apoptotic cells was detected, at which point TUNEL signal was observed across some 63% of the explant area (Figures 6E,K,N,O). This corresponded to roughly a halving of DAPI+ area between 2 and 4 days, in contrast to control explants, which approximately doubled in size over the same period (Figure 6P). These data reveal that SHH acts as a mitogen and survival factor, although the fact that SOX9+ area is reduced by 70% prior to any significant changes in proliferation and apoptosis (Figure 5A) indicates that SHH is also required for the formation of SOX9+ VZ progenitors. Similar to the telencephalon (Scott et al., 2010), this could occur through the induction of SOX9, or alternatively SHH may be required to maintain SOX2+ progenitors prior to SOX9 induction (Figure 6Q).

Addition of DAPT to pHyp explants led to reduced proliferation. EdU incorporation was significantly reduced at 2 days, and at 4 days was virtually abolished; and a 60% decrease in pH3+ area was seen at 4 days (Figures 6C,F,M,N). DAPT also significantly increased apoptosis, and this effect too was detected earlier than that of cyclopamine: TUNEL-positive area peaked at 45% at 2 days, subsiding to 25% at 4 days (Figure 6I,L,O). Accordingly, explants were 77% smaller than control after 2 days, with some recovery evident at 4 days (Figure 6P). Changes in proliferation, apoptosis, and explant area therefore occurred earlier in DAPT treated explants than they did after cyclopamine administration.

The rapid slowdown in proliferation in Notch-inhibited samples, evident after 2 days of culture, squares with the premature generation of large numbers of P57+/POMC+ neurons and their associated cell cycle exit, although a positive effect of Notch on progenitor renewal *per se* cannot be excluded (Figures 5A,B). A further effect on survival is also indicated by the results of the TUNEL assay (Figures 5I,L,O). Based on the VZ expression of *HES5*, the loss of SOX2 and SOX9 with DAPT treatment, and the widespread expression of neurogenic markers in DAPT-treated explants (Figures 4d””, 5C–J), we predict that SOX9+ progenitors are undergoing apoptosis in DAPT-treated explants.

In summary, inhibiting either SHH or Notch signalling led to reduced proliferation and high apoptosis, but these effects were detected earlier in Notch-inhibited explants. As SOX9+ area was strongly reduced before any differences in cell cycle, cell death, or explant size were evident, we infer that SHH is independently required for normal SOX9 expression (Figure 6Q).

### Interaction between SHH and Notch pathways

**FIGURE 7:**
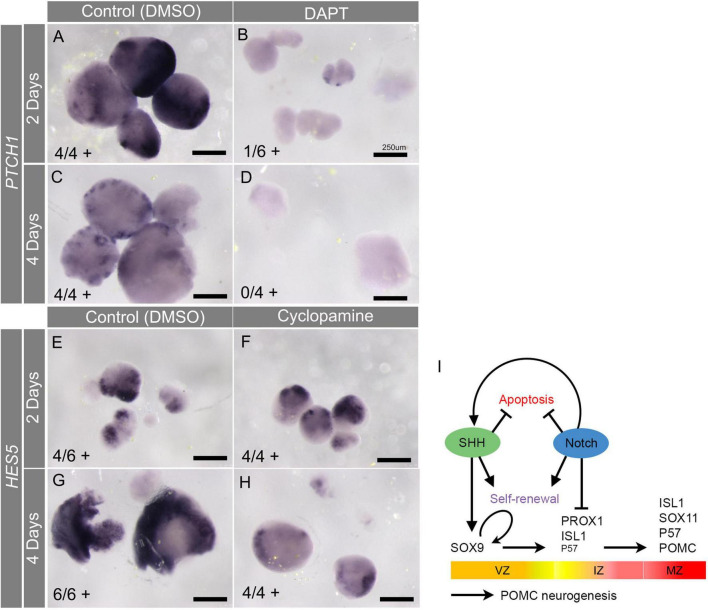
**(A–H)** Results of *ex vivo* culture experiments on pHyp explants cultured in the presence of DMSO **(A,C,E,G)**, the Notch inhibitor DAPT **(B,D)**, or the SHH inhibitor cyclopamine for 2 **(A,B,E,F)** or 4 **(C,D,G,H)** days. Explants were processed by colourimetric *in situ* hybridisation for *PTCH1* **(A–D)** or *HES5* **(E–H)** mRNA. Numbers in brackets indicate the number of explants staining positively, and the total number of explants analysed. **(I)** Schematic summarising transcription factor expression over the course of POMC neurogenesis. Proposed roles of SHH signalling in inducing SOX9 expression, and of Notch signalling in inhibiting differentiation of IZ PROX1+ cells, are indicated. Hypothetical scenario is indicated, whereby SHH inhibits apoptosis directly, and Notch indirectly, by facilitating SHH signalling. Curved arrow indicates self-renewal of SOX2+/SOX9+ VZ progenitor cells. Horizontal arrows indicate temporal progression of POMC neurogenesis. VZ, ventricular zone; IZ, intermediate zone; MZ, mantle zone.

Considering the *ex vivo* data as a whole, some overlap was apparent in the effects of SHH and Notch inhibition on SOX9+ progenitors (reduced), proliferation (reduced), and apoptosis (increased). Loss of Notch signalling therefore partially phenocopies loss of SHH. Intriguingly in this regard, recent studies in chick and mouse have suggested that Notch may help to maintain SHH signalling in the spinal cord and rostral ventral forebrain (Stasiulewicz et al., 2015; Marczenke et al., 2021). To assist in the interpretation of our results, we therefore asked whether inhibiting Notch would reduce SHH signalling in cultured pHyp explants. In line with this prediction, DAPT treatment of explants reduced *PTCH1* staining at both 2 and 4 days (Figures 7A–D), indicating that the Notch pathway supports SHH signalling in the hypothalamic VZ. We next considered whether SHH might also promote Notch signalling in a mutually reinforcing feedback loop by investigating whether cyclopamine would reduce *HES5* expression. In this case, *HES5* levels were similar in control and cyclopamine-treated samples (Figures 7E–H), indicating that while Notch is required to maintain SHH signalling, the reverse is not true.

This result suggests that the loss of SOX9+ cells in DAPT-treated explants may be secondary to a reduction in SHH signalling, in addition to the depleting effect of premature differentiation. It furthermore suggests that any direct effects of Notch withdrawal, in reducing progenitor self-renewal and promoting apoptosis, may be augmented by the indirect effects of losing SHH signalling (Figure 7I). Considered together, the results presented herein suggest a role for SOX9 in POMC neuronal differentiation, and suggest that complex interactions between the SHH and Notch pathways are required to establish the correct balance between progenitor emergence, survival, and differentiation.

## Discussion

Our immunohistochemistry analysis of the developing chick hypothalamus suggests that the TFs SOX2, SOX9, PROX1, ISL1, and SOX11 are sequentially expressed during the course of POMC neurogenesis. Results from *ex vivo* explant cultures also suggest that SHH is required for SOX9+ VZ cells, which arise from SOX2+ neuroepithelial cells. Notch is required to limit the initial differentiation of SOX9+ VZ cells toward PROX1+ IZ early neurogenic cells and, eventually, POMC neurons. Both factors promote progenitor proliferation, and cell survival.

### Sequential expression of TFs in the developing tuberal hypothalamus

We have provided evidence for the sequential expression of SOX2, SOX9, PROX1, ISL1, and SOX11 in POMC neurogenesis. This is supported by: the VZ/IZ/MZ location of these proteins, which closely correlated with the timing of their expression along a tuberal pseudotime trajectory from a chicken hypothalamic scRNA-seq dataset (Kim et al., 2022); the protein co-expression of SOX2/SOX9 and ISL1/SOX11; the protein and mRNA co-expression of SOX9/ISL1, PROX1/ISL1, PROX1/POMC and ISL1/POMC; and the mRNA co-expression of PROX1/ISL1/POMC.

Although we have shown co-expression of temporally overlapping pairs of TFs, we have not proven that these factors act in a continuous sequence along a single differentiation path. Tuberal hypothalamic neurogenesis encompasses the development of diverse neuronal types, and at present, it is not understood in detail at what point these divergent neurogenic pathways become distinct from one another. Even POMC is not restricted to a single hypothalamic lineage–although based on their number and high POMC expression levels, classical POMC/PRDM12-expressing ARC neurons likely represent the majority of POMC-expressing neurons detected in our study (Campbell et al., 2017; Yu et al., 2021).

The onset of SOX9 expression at the VZ follows SOX2 and coincides with the appearance of ISL1+ neurons (Figures 2B–J). This is consistent with the hypothesis that early NKX2.1+/SOX2+ neuroepithelial cells are pre-neurogenic, and that SOX9 expression may correspond to a temporal switch to a mature, neurogenic VZ in the SHH+ basal hypothalamus (Scott et al., 2010). The scRNA-seq tuberal pseudotime also suggested that SOX9 and ZEB1 initiate at similar times, and indeed, these factors co-localised in the VZ/IZ (Figures 2T–U”).

PROX1 has not previously been connected to the development of ARC POMC neurons, although various roles have been described in other regions of the nervous system. Consistent with its IZ location (Figures 3C,D), PROX1 controls the differentiation of spinal interneurons, and is required for neuronal traits and for cell cycle exit of the IZ cells (Misra et al., 2008; Lu et al., 2012; Kaltezioti et al., 2014). The fly PROX1 homologue Prospero both represses progenitor states and prevents cell cycle reentry of differentiating cells (Choksi et al., 2006). This raises the possibility of similar roles for PROX1 in ARC neurogenesis. Further studies will be needed to describe in detail the effects of PROX1 on ARC POMC+ neuron development.

### Possible role of TFs in promoting EMT-like cellular changes

The TFs studied in this work have a multitude of distinct cellular roles in multiple tissues, and each has also been linked to the regulation of EMT. In a classic developmental EMT context, SOX9 is a key regulator of neural crest development and of EMT itself (Akiyama et al., 2004; Cheung et al., 2005; Sakai et al., 2006). ZEB1 is one of a handful of core EMT-TFs which are thought, with some redundancy, to be involved in all known developmental EMT processes (Yang et al., 2020). PROX1 regulates cell adhesion and polarity of colon cancer cells, and promotes invasion of several tumour types (Dadras et al., 2008; Petrova et al., 2008; Elsir et al., 2012). ISL1 has been described as a key regulator of heart epicardium to myofibroblast EMT, promoting mesenchymal features and under negative regulation by microRNA-31 (Brønnum et al., 2013). SOX11 promotes breast cancer cell migration and activates SLUG, leading to EMT (Shepherd et al., 2016; Xiao et al., 2020).

The context-dependent abilities of these TFs to regulate aspects of EMT, in addition to an array of other molecular functions, therefore highlights the question of whether some or all of them are directly involved in the changes in polarity, adhesion, morphology, and migration that accompany hypothalamic neurogenesis. Such roles have been demonstrated for FOXP TFs (adhesion/polarity) and the proneural factors ASCL1 and NEUROG2 (migration) in spinal cord and cerebral cortex, respectively (Heng et al., 2008; Pacary et al., 2011; Rousso et al., 2012). Seen in this light, it is notable that the onset of SOX9 and ZEB1 expression is coincident with the onset of neurogenesis. ISL1 partially overlapped with BLBP in the IZ (Figures 1X–Y”), suggesting that it could be expressed as radial glia start to undergo the EMT-like changes associated with neurogenesis (Singh and Solecki, 2015). These observations would therefore seem consistent with the possibility of these TFs acting to suppress epithelial features and/or promote mesenchymal properties.

### SHH and Notch effects on POMC-expressing neurogenesis

To investigate the roles of SHH and Notch signalling on POMC neurogenesis we conducted explant studies using their respective inhibitors cyclopamine and DAPT. Notably, both treatments resulted in reduced proliferation and high levels of apoptosis, although in the case of cyclopamine, these effects were not significant until 4 days of culture. Both treatments also led to the loss of SOX2+/SOX9+ VZ cells, but their effects on neuronal differentiation were opposite: all IZ/MZ-expressed factors were increased following DAPT administration, but–with the exception of PROX1–decreased after 4 days of cyclopamine treatment (Figures 5A–J). This suggests that while both factors are required by the SOX9+ VZ in the SHH+ basal hypothalamus, they may act at different levels.

SHH appears necessary for the generation of SOX9+ cells, inferred by the fact that this marker was greatly reduced before significant changes in proliferation or cell death were detected. We did not establish whether this is due to an effect on SOX2+ neuroepithelial cells or the transition to SOX2+SOX9+ VZ cells. However, a potential role for SHH in inducing the hypothalamic SOX9+ VZ is highlighted by the earlier finding that SHH-induced SOX9 is necessary and sufficient for inducing NSC of both dorsal telencephalon and spinal cord (Scott et al., 2010).

Meanwhile, our data indicates that Notch is required to limit the differentiation of SOX9+ progenitors toward POMC neurons (Figures 5A–J,e). Consistent with earlier work reporting inhibitory effects of Notch on hypothalamic neuronal differentiation (Aujla et al., 2013; Ratié et al., 2013; Ware et al., 2016), DAPT treatment of pHyp *ex vivo* in the present study led to increased numbers of early (IZ, PROX1+/ISL1) and late (MZ, ISL1+/P57+/SOX11+/POMC+) differentiating neurons, alongside a depleted pool of SOX9+ VZ progenitors. A further effect on late differentiation, for instance by repressing P57, also cannot be excluded (Zalc et al., 2014). Although the present study focussed exclusively on POMC neurogenesis, it is interesting to note that different neural subtypes may have differential requirements for Notch signalling. While conditional loss of Rbpjκ from the mouse hypothalamus led to increased generation of ARC POMC neurons (Aujla et al., 2013), ARC Kisspeptin-expressing neurons–some of which derive from POMC+ progenitors–are lost in the same model (Biehl and Raetzman, 2015).

SHH and Notch therefore have overlapping but distinct roles in hypothalamic POMC neurogenesis, cooperating to achieve the correct balance between self-renewal and differentiation of progenitors. Mounting evidence suggests that Notch may support SHH signalling in the spinal cord and basal hypothalamus (Stasiulewicz et al., 2015; Marczenke et al., 2021), suggesting that some of their common effects may be mediated by signal crosstalk. We now report that Notch signalling is required for PTCH1 expression in cultured hypothalamic explants (Figures 7A–D), suggesting that the SHH and Notch pathways synergise to maintain the SOX9+ hypothalamic VZ. Therefore, some of the observed effects of Notch inhibition may be secondary to loss of the SHH response. Notably, however, we did not find evidence that SHH was reciprocally required for Notch activity (Figures 7E–H).

Complex interactions between TFs and signalling pathways occur as neurogenesis proceeds, and future studies will be required to unpick these in detail. It is possible that PROX1 may promote the progression of neurogenesis by suppressing NOTCH1 receptor expression as seen in the mouse and chick neural tube (Kaltezioti et al., 2010). Following from this, and our description of PROX1 as an early expressed IZ gene, one hypothetical possibility is that PROX1 is sensitive to early changes in Notch signalling, and helps to ensure a robust loss of signal during early differentiation. At this stage it is not apparent why PROX1, uniquely out of the markers studied, was spared in cyclopamine-treated explants (Figure 5D), but it is possible that PROX1 also operates in another pathway, differentially affected by SHH.

Interestingly, one mechanism by which Notch supports neural progenitors in the zebrafish hindbrain is by maintaining apicobasal polarity and thus the epithelial state (Ohata et al., 2011). Thus, in addition to transcriptional effects on *HES/HEY* and downstream genes, Notch may also directly promote epithelial features, its loss therefore contributing to EMT-like changes. Altogether, the balanced activities of SHH, Notch, and the TFs studied likely play varied and interdependent roles in regulating the changes in adhesion, polarity, morphology and migration that accompany POMC neurogenesis.

## Conclusion

We have investigated the expression of TFs with the context-dependent ability to regulate EMT during pre- and early neurogenic stages in the chicken hypothalamus, with an emphasis on ARC POMC neurogenesis. Our findings suggest that SOX2, SOX9, PROX1, ISL1, and SOX11 are sequentially expressed over the course of POMC neurogenesis. SHH and Notch pathways are both required for the maintenance of the SOX9+ neurogenic VZ, with SHH likely promoting its generation and Notch limiting its differentiation toward POMC neurons. Further studies will be necessary to understand the role of these TFs in regulating EMT-like aspects of neurogenesis and the interplay of SHH, Notch and TFs in controlling ARC POMC neurogenesis. An improved understanding of how anorexigenic ARC POMC+ neurons are generated is likely to aid efforts to understand the molecular mechanisms helping to prevent obesity and metabolic syndrome.

## Data availability statement

The datasets presented in this study can be found in online repositories. The names of the repository/repositories and accession number(s) can be found below: https://www.ncbi.nlm.nih.gov/search/all/?term=GSE171649.

## Ethics statement

This animal study was reviewed and approved by Tokyo Medical University and The University of Sheffield.

## Author contributions

KO: experimental design. EP, EM, and KO: writing and editing the manuscript. All authors contributed to the article and approved the submitted version.

## Supplementary material

Supplementary Figure 1 **(A–D)** Prospective hypothalamic (pHyp) explants cultured in the presence of DMSO **(A,C)** or the SHH inhibitor cyclopamine **(B,D)** for 2 days **(A,B)** or 4 days **(C,D)** and processed by colourimetric *in situ* hybridisation for *PTCH1*. **(E–H)** pHyp explants cultured in the presence of DMSO **(E,G)** or the Notch inhibitor DAPT **(F,H)** for 2 days **(E,F)** or 4 days **(G,H)** and processed by colourimetric *in situ* hybridisation for *HES5*. Numbers in brackets indicate the number of explants staining positively, and the total number of explants analysed. Coloured charts represent the number of explants staining positively (purple) or negatively (white) for the marker of interest, in control (left) and treatment (right) conditions.
