# Supplementary figures and images for: SHH and Notch regulate SOX9+ progenitors to govern arcuate POMC neurogenesis
Source: Front Neurosci. 2022 Aug 11;16:855288. doi: 10.3389/fnins.2022.855288 (PMC9404380; doi:10.3389/fnins.2022.855288)

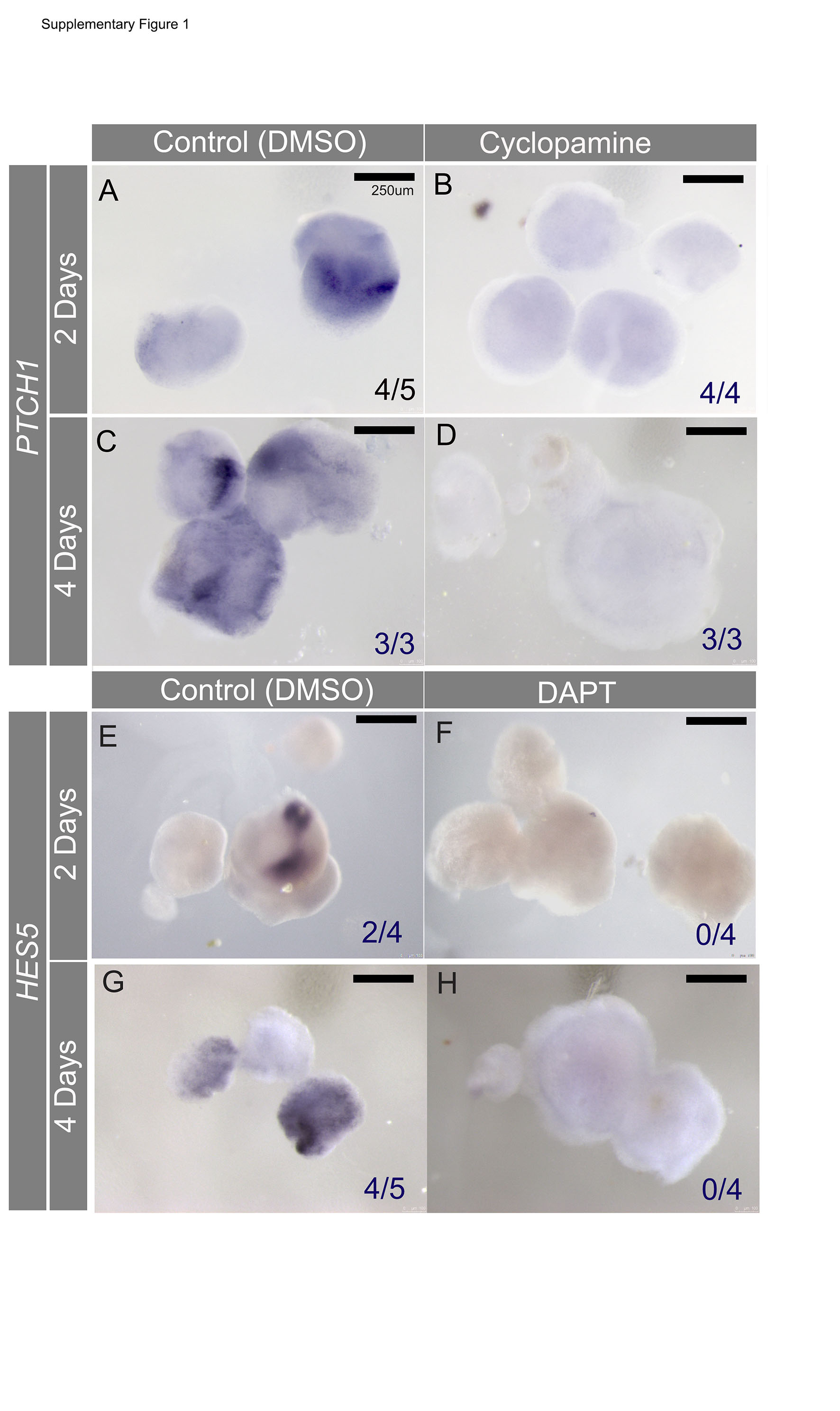

Supplement: Supplementary Figure 1 — (A–D) Prospective hypothalamic (pHyp) explants cultured in the presence of DMSO (A,C) or the SHH inhibitor cyclopamine (B,D) for 2 days (A,B) or 4 days (C,D) and processed by colourimetric in situ hybridisation for PTCH1. (E–H) pHyp explants cultured in the presence of DMSO (E,G) or the Notch inhibitor DAPT (F,H) for 2 days (E,F) or 4 days (G,H) and processed by colourimetric in situ hybridisation for HES5. Numbers in brackets indicate the number of explants staining positively, and the total number of explants analysed. Coloured charts represent the number of explants staining positively (purple) or negatively (white) for the marker of interest, in control (left) and treatment (right) conditions. [file Image_1.JPEG]
